# Exercise and hypertrophic cardiomyopathy: Two incompatible entities?

**DOI:** 10.1002/clc.23343

**Published:** 2020-02-12

**Authors:** Joyee Basu, Aneil Malhotra, Michael Papadakis

**Affiliations:** ^1^ Cardiac Risk in the Young Clinical Research Fellow, Cardiology Clinical Academic Group St George's University of London London UK; ^2^ Division of Cardiovascular Sciences The University of Manchester Manchester UK

**Keywords:** cardiac hypertrophy, cardiac rehabilitation, cardiomyopathy, sports medicine

## Abstract

A greater understanding of the pathogenic mechanisms underpinning hypertrophic cardiomyopathy (HCM) has translated to improved medical care and better survival of affected individuals. Historically these patients were considered to be at high risk of sudden cardiac death (SCD) during exercise; therefore, exercise recommendations were highly conservative and promoted a sedentary life style. There is emerging evidence that suggests that exercise in HCM has a favorable effect on cardiovascular remodeling and moderate exercise programs have not raised any safety concerns. Furthermore, individuals with HCM have a similar burden of atherosclerotic risk factors as the general population in whom exercise has been associated with a reduction in myocardial infarction, stroke, and heart failure, especially among those with a high‐risk burden. Small studies revealed that athletes who choose to continue with regular competition do not demonstrate adverse outcomes when compared to those who discontinue sport, and active individuals implanted with an implantable cardioverter defibrillator do not have an increased risk of appropriate shocks or other adverse events. The recently published exercise recommendations from the European Association for Preventative Cardiology account for more contemporary evidence and adopt a more liberal stance regarding competitive and high intensity sport in individuals with low‐risk HCM. This review addresses the issue of exercise in individuals with HCM, and explores current evidence supporting safety of exercise in HCM, potential caveats, and areas of further research.

## 1 INTRODUCTION

The benefits of exercise on cardiovascular health and all‐cause mortality are well established.^1^, ^2^, ^3^, ^4^, ^5^, ^6^, ^7^, ^8^, ^9^ However, studies have demonstrated a nearly threefold increased risk of sudden cardiac death (SCD) in young athletes with underlying cardiovascular conditions when compared to sedentary individuals.^10^ Hypertrophic cardiomyopathy (HCM), first described by Teare^11^ is a genetic disorder, characterized by the presence of increased left ventricular wall thickness (≥15 mm) that is not solely explained by abnormal loading conditions.^12^ Historically, HCM was considered to be the leading cause of SCD in athletes.^13^, ^14^, ^15^ International recommendations therefore stood firm on the exclusion of athletes with HCM from most competitive sports.^16^, ^17^ Although scientific bodies approach the issue of participation of nonathletic patients with HCM in recreational sport in a more liberal fashion, guidance regarding safe levels of exercise remains vague.^18^ Consequently, patients adopt a sedentary lifestyle, with significant impact on their physical and psychological well‐being.^19^, ^20^, ^21^, ^22^, ^23^, ^24^, ^25^, ^26^

HCM is the most common ICC encountered in clinical practice and is thought to affect around 1 in 500 adults.^27^, ^28^, ^29^, ^30^ Recent data suggest that the prevalence may even be as high as 1 in 200.^31^ Extrapolating such figures indicate that approximately 120 000 people in the United Kingdom and 20 million people worldwide are affected,^32^ representing a significant global burden.^33^ The implementation of primary and secondary preventative strategies; preparticipation screening,^34^, ^35^, ^36^, ^37^ mandated familial evaluation following SCD or diagnosis of ICC in a family,^38^ education around cardiopulmonary resuscitation and automated external defibrillators^39^, ^40^ have resulted in an expansion of the HCM population. Furthermore, advances in management such as implantable cardioverter defibrillator (ICD) implantation have led to a reduction in mortality.^41^ As a result, HCM mortality rates are now comparable to the general population.^42^ Consequently, the need to reduce cardiovascular risk over a normal lifespan becomes a priority in these individuals.

**Figure 1:**
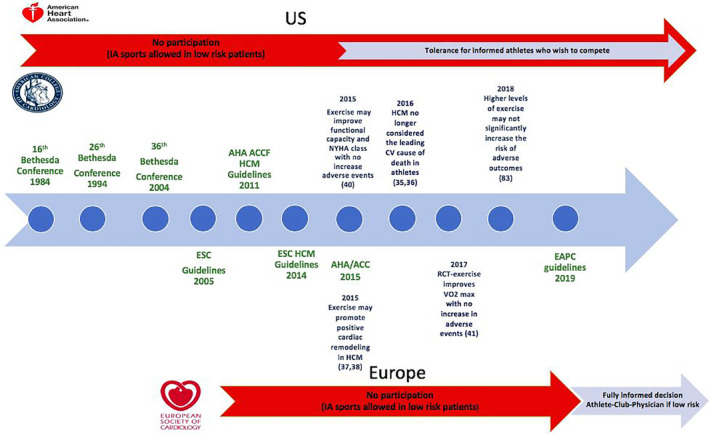
Key studies of exercise in HCM and the resultant evolution of exercise recommendations

The risk of SCD during exercise in individuals with HCM, may not be as high as initially perceived. Recent postmortem studies have demonstrated that HCM accounts for a much smaller proportion of SCD in athletic individuals.^43^, ^44^ Moreover, recent studies of cardiac rehabilitation programs in older patients with HCM in their sixth decade of life, suggest that exercise in moderation may be safe.^45^, ^46^ Murine models and clinical studies in athletes with HCM suggest that exercise may even lead to favorable cardiac remodeling.^47^, ^48^ In a small study of lifelong competitive athletes with HCM there was no difference in terms of outcomes in athletes who adopted a more sedentary lifestyle compared to those who continued competitive sport.^49^ The shifting opinion regarding safety of exercise in HCM is reflected in the most recent European Association of Preventative Cardiology (EAPC) guidelines which support a more liberal approach towards sports participation in low risk individuals with HCM.^50^ Figure 1 demonstrates the evolution in exercise recommendations from the European Society of Cardiology (ESC) and American Heart Association/American College of Cardiology (AHA/ACC) alongside key contributory studies of exercise in HCM. There still, however, remains intense debate among specialists as to whether the pendulum has now swung too far in the other direction.

### 1.1 SCD and HCM

Although the benefits of exercise are compelling, SCD is the leading medical cause of death in athletes.^10^, ^13^, ^14^, ^51^ The incidence is between 1 and 2 per 100 000 person years^10^, ^13^, ^14^, ^51^, ^52^, ^53^ and may be as high as 6.8 per 100 000 person years among adolescent athletes.^54^ Maron et al demonstrated that HCM was the leading cause of SCD in US athletes accounting for up to 36% of deaths.^13^, ^14^, ^15^ In a prospective cohort study of SCD in young individuals in the Veneto region of Italy the incidence of SCD was 2.3 per 100 000 per year in athletes compared to 0.9 per 100 000 per year in nonathletes. The higher risk of sudden death in athletes was attributable to underlying cardiovascular conditions, therefore suggesting a 2.8‐fold increased risk in athletes with a predisposing cardiac pathology.^10^ These studies have largely formed the basis for conservative exercise recommendations in individuals harboring serious cardiac conditions such as HCM. The 2005 ESC guidelines^16^ and the 2015 AHA guidelines^17^ restrict those individuals with a definite diagnosis of HCM from competitive sport with the exception of low intensity sports (bowling, billiards, curling, riflery, and golf). Although the same scientific bodies approach the issue of participation of HCM patients in recreational sport in a more moderate way, the guidance regarding the intensity of exercise is vague.^18^

### 1.2 The impact of conservatism

Results from the Italian data^55^ have been presumptively extrapolated to all ICC and all levels of exercise. As a result, physicians often encourage patients to adopt a sedentary lifestyle or provide vague and impractical advice with regards to exercise.^21^, ^23^ This practice is compounded by the paucity of data relating to the beneficial effects in this subset of patients with ICC and most importantly, the safety of a structured exercise program. Presently, 55% of patients fail to meet the minimum physical activity recommendations.^19^, ^20^ A sedentary lifestyle cultivates obesity and increases cardiovascular risk. In large cohort studies, up to 70% of individuals with HCM were found to be preobese or obese.^26^ Obesity in HCM leads to a significant increase in left ventricular mass, obstructive physiology, worsening heart failure symptoms as well as an increased likelihood of atrial fibrillation when compared to nonobese individuals.^25^, ^26^ Moreover, a sedentary lifestyle promotes the development of additional risk factors for atherosclerosis and coronary artery disease (CAD), both associated with excess mortality. In a cohort of 425 patients with HCM, hypertension was present in 58% of black patients and 32% of white patients, and was an independent risk factor (HR 2.02, 95% CI 1.05 to 3.88, *P* = .036) for a composite outcome of cardiovascular death, cardiac arrest or appropriate ICD therapy.^56^ Sorajja et al demonstrated that individuals with HCM and severe CAD were at greater risk of death, compared to those with mild to moderate or no CAD, and their risk far exceeded historical death rates of CAD patients with normal left ventricular function.^24^ Additionally, the psychological impact of a diagnosis of HCM should not be underestimated. In both competitive, as well as recreational sportsmen and women, sport restrictions can lead to anxiety and significantly worsen social functioning and stress management.^19^, ^21^, ^22^, ^23^

### 1.3 Are we overestimating the risk?

The risk of SCD during exercise in HCM may not be as high as initially thought. Although, Maron et al cited HCM as the most common cause of cardiovascular death in athletes,^13^, ^14^, ^15^ other studies have shown contrary evidence. Contemporaneous reports from Italy demonstrated that coronary artery anomalies, arrhythmogenic right ventricular cardiomyopathy (ARVC), and premature cardiovascular disease were the most common causes of SCD.^10^ In a study of consecutive cases of athletes referred to a national center for cardiovascular pathology^43^ sudden arrhythmic death syndrome was the most common cause of death, in 42% of cases, while HCM contributed to only 6% of the deaths. Similar findings have been reported in unselected cohorts of U.S. collegiate athletes and young sudden deaths in Australia.^44^, ^53^ In addition, the association between HCM and SCD during exertion is based predominantly on circumstantial evidence and contrasts to the mounting evidence of adverse outcomes in individuals with ARVC/carriers who participate in regular moderate or intense exercise.^57^, ^58^ One, however, should exercise caution as we lack risk stratification protocols for HCM in athletes and extrapolating data from predominantly sedentary cohorts may underestimate the risk. In a large study of adolescent football players who underwent cardiac screening, three of the eight deaths were due to HCM, two of whom had been identified during screening and continued to exercise.^54^

### 1.4 Potential beneficial effects of exercise in hypertrophy cardiomyopathy

Physical inactivity may exacerbate pathological processes already limiting exercise capacity. Exercise intolerance in individuals with HCM is mediated by a number of factors. These include a predisposition to arrhythmias including atrial fibrillation (AF)^59^, ^60^ and exercise‐induced arrhythmias.^61^ Pathological structural and vascular changes in the heart may give rise to myocardial ischaemia,^62^ diastolic dysfunction^63^, ^64^ and left ventricular outflow tract obstruction.^65^ Individuals may demonstrate an inability to respond to increases in activity due to an abnormal blood pressure response^66^ and/or chronotropic incompetence.^67^ Peripheral deconditioning is also a major contributor to reduced exercise tolerance. Exercise may help offset some of these limiting factors. In patients with HF, improvements in exercise capacity have been attributed to peripheral adaptation, including reduction in endothelial dysfunction in the skeletal muscle vasculature as well as increased oxidative capacity.^68^, ^69^ Exercise may also increase stroke volume and favorably remodel cardiac dimensions.^70^ Exercise interventions have demonstrated beneficial outcomes in patients with HF^71^, ^72^ reducing mortality and hospital admissions. Exercise may also exert a beneficial effect on diastolic filling and improve exercise capacity in patients with HF with preserved ejection fraction.^72^ The effects on diastology suggest that exercise may benefit individuals with established HCM in whom diastolic dysfunction is a significant contributor to exercise limitation. This notion is supported by a study in 106 athletes with HCM which showed that athletes exhibited normal or supranormal indices of diastolic function when compared to sedentary patients.^47^ Moreover, experimental studies in murine models of HCM demonstrate that exercise may halt or even reverse the cardiomyopathic process.^48^ Mice with HCM initiated exercise, in a voluntary cage wheel, at either 2 or 6 months. Pathological evaluation at 8 months of those who began exercising prior to disease expression, demonstrated inhibition of myocardial fibrosis and disarray, whereas older mice showed only reversal of disarray. Exercise also prevented the induction of hypertrophic markers and favorably affected apoptotic pathways in both groups. Notably, there was no difference in mortality between mice with HCM and non‐transgenic mice.

### 1.5 Cardiac rehabilitation in the general HCM population

Two studies in HCM patients suggest that exercise may improve fitness without a concomitant increase in arrhythmic burden. In a prospective nonrandomized study of 20 symptomatic patients (mean age 62 years), exercise intensity was increased from 50% to 85% of an individual's heart rate reserve (HRR). Functional capacity, assessed by a graded exercise test, improved from 4.7 to 7.2 METS (*P* = .01). NYHA functional class also improved from baseline by ≥1 grade in 10 patients (50%).^45^ The most recent randomized controlled trial, RESET‐HCM,^46^ assigned patients (mean age 50 years) to 16 weeks of an unsupervised home‐based moderate‐intensity exercise training program (*n* = 67) or usual activity (*n* = 69). Exercise intensity in the intervention group increased from 60% to 70% of the HRR. Mean peak oxygen consumption improved significantly (*P* = .02) in the exercise training group. There were no adverse events in either study. These studies demonstrate that exercise benefits the cardiovascular fitness of HCM patients and may be safer than initially anticipated.

It is important to note that these studies were not powered for safety. In the RESET‐HCM trial, although there was no death, aborted SCD, ICD shocks, or sustained ventricular tachycardia (VT), the authors reported that several participants experienced symptomatic nonsustained VT (NSVT). One patient who had previously had NSVT, experienced a further 30 s before starting the exercise program, while another patient (who had an ICD) demonstrated an increase in burden of symptomatic NSVT. A third patient experienced NSVT de novo 1 hour after exercising. All three individuals were withdrawn from the study. Although occurrence of nonfatal arrhythmias was not significantly different between groups, these episodes of symptomatic arrhythmia warranted ICD implantation, and raised concerns of further adverse events with continuation of exercise. With this in mind, careful consideration should be given to an individual's SCD risk score prior to provision of exercise recommendations. This also highlights the importance of careful monitoring of these individuals. Further work is needed in larger cohorts and younger age groups with the adoption of more vigorous exercise regimes.

### 1.6 Athletes with HCM

The recent position statement from the Sport Cardiology Section of EAPC recommends that participation in intense exercise or competitive sport may be considered after comprehensive clinical evaluation in asymptomatic adults, with low ESC‐SCD risk score and a mild HCM phenotype.

This excludes those sports where syncope may cause harm or death.^50^ These changing opinions are particularly pertinent to those individuals who continue to compete or exercise above recommendations provided by their physicians.^73^ Data supporting the change in recommendations stem from the clinical profiling of athletes with HCM who are capable of competing at an elite level. Sheikh et al compared 106 elite athletes with HCM to 101 sedentary individuals with HCM. Athletes demonstrated a milder phenotype with regard to LVH, and a third demonstrated the apical variant of HCM, indicating a lower risk of SCD.^47^ Retrospective data from competitive athletes with HCM have also shown that higher levels of exercise may not significantly increase the risk of adverse outcomes. Results from a multinational, prospective ICD registry of 440 athletes with cardiovascular disease (17% with HCM), participating in high risk and organized sports, demonstrated no deaths related to arrhythmia, no aborted SCD or injury following syncope or shock during sport, during 44 months follow‐up period.^74^ In the original registry athletes with HCM demonstrated the lowest number of VT/VF shocks during competition/practice *n* = 1 (2%), in clear contrast to ARVC which was associated with increased risk of ICD shock during competition or practice.^75^

Pelliccia et al^76^ compared 15 athletes with HCM who continued to engage in regular exercise or competitive sport to athletes who had suspended exercise. Athletes had been engaged in sporting activities for a mean of 15 ± 8 years at a level equivalent to regional or above. Only two of these individuals were deemed to be high risk according to the ESC SCD risk calculator, two were intermediate risk and the remainder were low risk. None of these individuals had an ICD. Over the course of 9 ± 6 years of follow‐up only an amateur tennis player suffered a cardiac arrest while walking. In terms of symptoms seven individuals experienced these, the most significant being syncope (*n* = 3). The event and symptom rate were not significantly different between the two groups.

Although Pelliccia's group^76^ present optimistic findings there are several issues to be mindful of. We know that the risk of SCD is influenced by an athlete's demographics and sporting discipline. Higher risk individuals include males, adolescents, black athletes, and those competing in high intensity start‐stop sports such as football and basketball.^13^, ^14^, ^15^, ^54^ In this study, the majority of these athletes were male and Caucasian, therefore the results do not account for ethnic variation in SCD. The sample size was small, with relatively short follow‐up and therefore not powered to detect a significant difference in outcomes, particularly given the low‐event rate of SCD. Additionally, the population may not reflect the consideration that should be given to risk according to age. This was highlighted in a recent study of outcomes in competitive adolescent football players who underwent cardiac screening.^54^ Of the eight SCDs recorded, three were due to HCM, of which two had been identified and were advised not to continue playing competitively.

These findings highlight that estimation of the risk of an event is not an easy task. Although the great majority of individuals with HCM are expected to have a normal lifespan^41^, ^42^ the risk of SCD appears to vary with age. The highest risk is conferred to young individuals,^77^, ^78^, ^79^ with a notable decrease in risk above the age of 60 years.^80^ In the absence of previous hemodynamically compromising VT or ventricular fibrillation, an individual's risk may be calculated using the ESC‐SCD risk score calculator.^12^ However, the timing of adverse events is not predictable. The heterogeneity of outcomes is highlighted in studies of ICD therapies. Maron et al^81^ demonstrated that the interval between implantation and discharge varied widely, even by up to a decade.^82^ In secondary prevention cases, individuals may not experience further events for up to 30 years.^83^ Longer term follow‐up is required and most importantly considerations such as age, gender, ethnicity, sporting discipline, and ESC‐SCD risk score should be factored into advice regarding an individual's exercise recommendations.

### 1.7 Current practice and future perspectives

**Figure 2:**
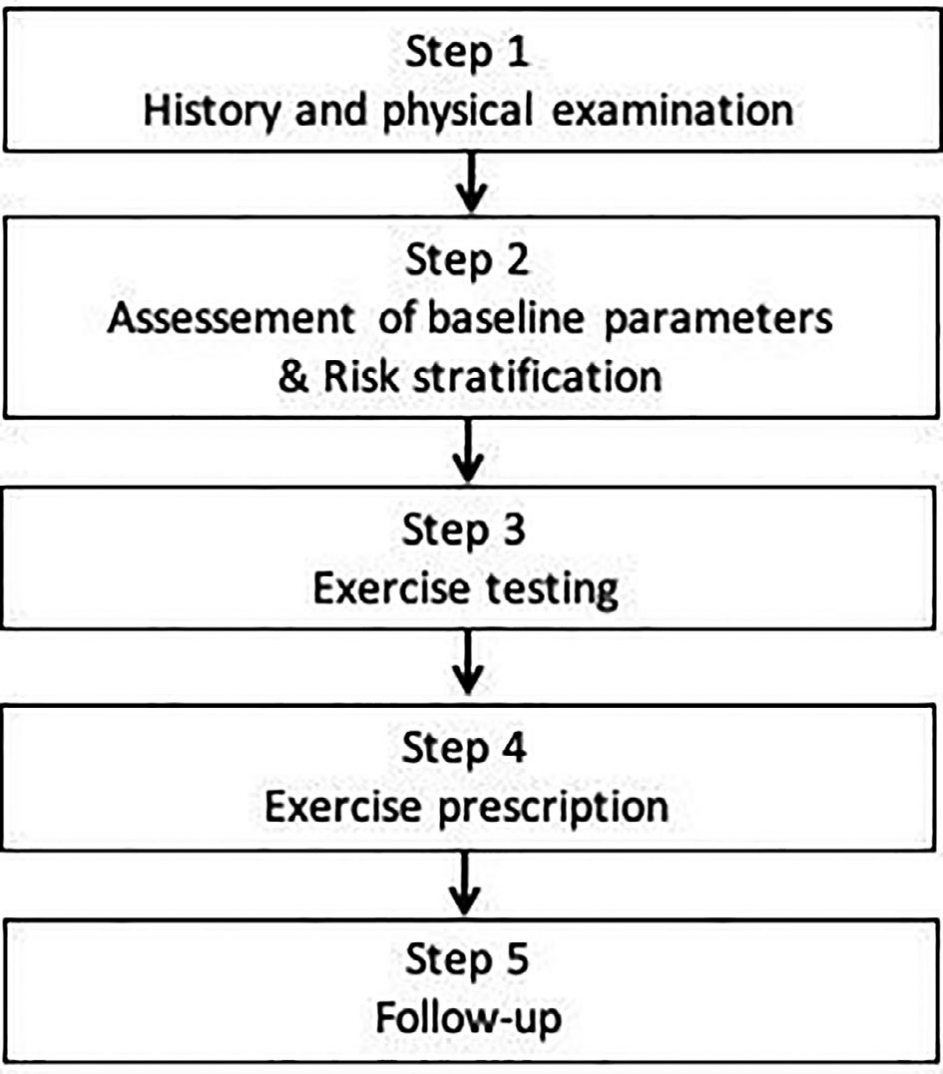
Proposed approach to exercise prescription

Based on the best available evidence, it seems reasonable to offer all patients with HCM a comprehensive evaluation (Figure 2). This will allow for a more individualized approach guided by the patient's risk, symptoms, and baseline fitness. The exercise prescription should be specific and abide by the “FITT” principle (frequency, intensity, time [duration], type of exercise).^84^ Unless significant concerns exist, the exercise prescription should satisfy current WHO recommendations for physical activity.^85^ Maximal intensity should not exceed 70% of HRR, which approximately equates to 80% of maximal predicted HR or 14 to 16 of the Borg scale. Individuals at high risk of SCD, limited by symptoms or with low baseline fitness should be optimized on medical therapy and commenced at a lower exercise level which could then be gradually increased based on their response. It seems reasonable that for individuals who would qualify for competitive sport, based on the Sport Cardiology Section of EAPC recommendations,^42^ not to restrict the intensity level of recreational exercise. For competitive athletes, recommendations should be guided by the Sport Cardiology Section of EAPC recommendations.^42^ Ultimately, decisions relating to exercise prescription should take into consideration the wishes of a well‐informed patient.

Future research relating to competitive athletes should focus on large, multicenter registries as the option of randomized studies does not seem pragmatic. Large, randomized, multicenter studies, however, are possible in the general HCM population and should explore benefits and safety of different exercise regimes including higher intensity programs and high intensity interval training, which are likely to be more appealing to the expanding population of younger patients.

## 2 CONCLUSION

It is clear that we are in the early stages of gathering data on safety of exercise in HCM, but the advances in our understanding of the condition and its relation to exercise as a trigger of fatal arrhythmias should not be ignored. Previous consensus guidelines regarding exercise prescription were based on historical data and were overly restrictive. The new guidelines propose a less restrictive approach, under specific circumstances and after detailed discussion with the athlete and other stakeholders. Although this is a welcomed perspective, one must remain mindful that adequately powered, large cohort studies with long‐term follow‐up are currently lacking. Therefore, at present, an individualized rather than a one‐size fits all approach is preferable.

## CONFLICT OF INTEREST

The authors declare no potential conflict of interests.
